# FAP, CD10, and GPR77-labeled CAFs cause neoadjuvant chemotherapy resistance by inducing EMT and CSC in gastric cancer

**DOI:** 10.1186/s12885-023-11011-0

**Published:** 2023-06-05

**Authors:** Zehua Zhao, Yanmei Zhu

**Affiliations:** Department of Pathology, Dadong District, Affiliated Cancer Hospital of Dalian University of Technology (Liaoning Cancer Hospital and Institute, Cancer Hospital of China Medical University), No. 44 of Xiaoheyan Road, Shenyang, 110042 China

**Keywords:** CAF, Chemo-resistance, EMT, CSC, Gastric cancer

## Abstract

**Objective:**

A significant proportion of patients can not benefit from neoadjuvant chemotherapy (NCT) due to drug resistance. Cancer-associated fibroblasts (CAFs) influence many biological behaviours of tumors, including chemo-resistance. This study aims to explore whether CAFs expressing FAP, CD10, and GPR77 affect the efficacy of NCT and the prognosis of patients with gastric cancer, and its mechanism.

**Methods:**

One hundred seventy-one patients with locally progressive gastric adenocarcinoma who had undergone NCT and radical surgery were collected. Immunohistochemistry was used to detect the expression of FAP, CD10, and GPR77 in CAFs; the EMT markers (N-cadherin, Snail1, and Twist1) and the CSC markers (ALDH1, CD44, and LGR5) in gastric cancer cells. The χ^2^ test was used to analyze the relationship between the expression of CAF, EMT, and CSC markers and the clinicopathological factors, as well as the relationship between CAF markers and EMT, and CSC markers. Logistic regression and Cox risk regression were used to analyze the relationship between the expression of CAF, EMT, and CSC markers and TRG grading and OS; Kaplan-Meier analysis was used for survival analysis and plotting the curves.

**Results:**

The expression of CAF markers FAP, CD10, and GPR77 was closely associated with that of EMT markers; FAP and CD10 were closely related to CSC markers. In the univariate analysis of pathological response, CAF markers (FAP, CD10, GPR77), EMT markers (N-cadherin, Snail1, Twist1), and CSC markers (ALDH1, LGR5, CD44), were all closely associated with pathological response (all *p* < 0.05). Only Twist1 was an independent factor affecting pathological response in multifactorial analysis (*p* = 0.001). In a univariate analysis of OS, expression of FAP and CD10 in CAF, as well as expression of EMT biomarkers (N-cadherin, Snail1), were significant factors influencing patient prognosis (all *p* < 0.05). Multifactorial analysis revealed N-cadherin (*p* = 0.032) and Snail1 (*p* = 0.028), as independent prognostic factors affecting OS.

**Conclusion:**

FAP, CD10, and GPR77 labeled CAF subgroup may lead to NCT resistance and poor prognosis by inducing EMT and CSC of gastric cancer cells in locally advanced gastric cancer patients.

**Supplementary Information:**

The online version contains supplementary material available at 10.1186/s12885-023-11011-0.

## Introduction

Gastric cancer (GC) is the fifth most common cancer and the fourth leading cause of cancer death worldwide [1, 2]. In China, the incidence and mortality rates of gastric cancer are the 2nd and 3rd among malignant tumors, respectively [3]. More than 2/3 of patients have already become advanced gastric cancer (AGC) rather than early-stage GC at the time of consultation [4]. Neoadjuvant chemotherapy (NCT) can reduce the size of the lesion, eliminate metastases or micrometastases, and improve the survival rate. It can also clarify whether the lesion are effective to chemotherapy regimens and guide the postoperative treatment plan [5–7]. However, NCT did not give the expected results in all patients to whom it was applied [8, 9]. This suggests that a significant proportion of cases are resistant to chemotherapeutic agents, thus limiting the benefit from NCT.

In recent years, the role of the tumor microenvironment (TME) in tumor drug resistance has received increasing attention [10]. TME refers to the internal environment in which tumors develop. It includes cancer-associated fibroblasts (CAFs), adipocytes, endothelial cells, immune cells, and extracellular matrix (ECM) [11, 12]. CAF, as a major component of TME, affects many biological behaviors of tumors, including tumor invasion, metastasis, proliferation, and drug resistance [13, 14]. CAF is strongly heterogeneous and many biomarkers have been used to identify CAF. CAF subpopulations expressing different markers have different biological functions. FAP is a type II integral membrane protein that belongs to the family of membrane-bound serine proteases. It exhibits dipeptidyl peptidases and collagenase activities [15]. FAP^+^CAFs promote the progression of various cancers, including gastric cancer [16], non-small cell lung cancer [17], prostate cancer [18], esophageal adenocarcinoma [19], clear cell renal cell carcinomas [20], ovarian cancer [21], and high-grade invasive urothelial carcinoma of the bladder [22]. CD10 and GPR77 have also been found to be CAF markers. CD10, also known as common acute lymphoblastic leukemia antigen, belongs to the family of zinc-dependent type II metalloproteinases. It can degrade various bioactive peptides in the ECM [23]; GPR77 belongs to the family of non-G protein-coupled receptors [24]. In uroepithelial carcinoma, CD10^+^ CAF was significantly associated with adverse clinicopathological factors including lymph node metastasis, squamous differentiation, and tumor necrosis [25]. In addition, a study found that CAF subpopulations with high CD10 and GPR77 expression were also correlated with poor prognosis and chemoresistance in patients with breast cancer and lung cancer [26]. Do CAFs expressing FAP, CD10, and GPR77 affect the therapeutic effect of neoadjuvant chemotherapy and prognosis in patients with gastric cancer? It is still unclear.

CAF can affect tumor drug resistance through several mechanisms, such as epithelial to mesenchymal transition (EMT) and enhancement of tumor cell stemness. EMT refers to a biological procedure in which epithelial cells lose their normal epithelial traits and obtain a partially mesenchymal phenotype. The loss of the original normal structure of the cells combined with a decrease in intercellular adhesion makes them more invasive and at the same time resistant to drugs [27, 28]. This process is mainly mediated by EMT transcription factors, including the Snail superfamily and the Twist family. Tumor cells undergoing EMT acquire mesenchymal markers such as N-cadherin [29, 30]. Cancer stem cells (CSC) are a fraction of the population of stem cells in tumor tissue that can self-renew and differentiate into heterogeneous cancer cells, thus maintaining the malignant phenotype of tumor cells and making them drug-resistant [31]. Markers such as ALDH1, CD44, and LGR5 have been used for CSC identification [32–35]. CAF can promote EMT and maintain cancer cell stemness by secreting various cytokines, such as IL-6, IL-1α, and IL-1β [26, 36]. It is unclear whether CAF expressing FAP, CD10, and GPR77 can influence the NCT efficacy in gastric cancer patients by affecting EMT or stemness in gastric cancer cells.

In this study, we explore the relationship between CD10, GPR77, FAP-positive CAF and clinicopathological factors and chemotherapy resistance in gastric cancer patients who received NCT and surgical resection, and further analyze the correlation between CD10, GPR77, FAP positive CAF and EMT, stemness in cancer cells. We found that FAP, CD10, and GPR77 labled CAF subgroup may promote gastric cancer progression, lead to NCT resistance and poor prognosis by inducing EMT and CSC of gastric cancer cells. This study will provide a preliminary theoretical basis for further exploring the mechanism of CAF leading to NCT resistance.

## Material and methods

### Patients

Gastric cancer specimens undergoing NCT followed by gastric surgical resection from June 2015 to June 2018 in the Gastric Surgery Department of Liaoning Cancer Hospital were selected with the following inclusion criteria: patients with clinically diagnosed locally progressive gastric cancer (8th American Joint Committee on Cancer (AJCC) clinical stage: CT2N1M0-T4N3M0, TNM stages II and III); pathologically confirmed gastric adenocarcinoma prior to treatment; undergoing NCT and radical gastrectomy with or without postoperative treatment. Exclusion criteria: preoperative radiotherapy; history of residual gastric cancer; combined with other malignancies; 3 or more changes in neoadjuvant regimen; incomplete staging or treatment information; insufficient sections or wax blocks to assess markers. After inclusion and exclusion criteria, 171 cases were available for our pathological analysis.

All patients were followed up every 3 months for the first 3 years; every 6 months for the next 3 years; and annually after that. Overall survival (OS) used in the statistics refers to the time from definitive diagnosis to death from any cause or to the last date of follow-up. This study was performed following the Declaration of Helsinki and approved by the Ethics Committee of Liaoning Cancer Hospital and Institute (20220306G).

### Pathological response assessment

The response of the selected tissues to chemotherapy was assessed by two senior pathologists using a double-blind method according to the Mandard system for grading the histological regression of the primary tumor: TRG 1 for complete regression, i.e. complete fibrosis with no evidence of residual tumor; TRG 2 for minimal residual tumor cells with fibrosis as the main component; TRG 3 for fibrosis and residual tumor, dominated by fibrotic components; TRG 4 for fibrosis and residual tumor, dominated by tumor; TRG 5 for no evidence of regression with extensive residual tumor. Predominantly. Other histopathological features were reassessed in the course of the evaluation. When disagreements between pathologists arose, the agreement was achieved by joint review and discussion via multiple-heads microscopy.

### Immunohistochemical staining

Immunohistochemical staining method was used to explore the expression of biomarkers including CAF markers (CD10, GPR77, and FAP), EMT markers (N-cadherin, Snail1, and Twist1), and CSC markers (ALDH1, CD44, and LGR5). Paraffin-embedded samples were sliced at 4 μm thickness. Endogenous peroxidase blockers (Fuzhou Maixin Biotechnology Development Co., Ltd., China) were used to block endogenous peroxidation, normal non-immune goat serum (Fuzhou Maixin Biotechnology Development Co., Ltd., China) was used for blocking, ready-to-use immunohistochemistry EliVisionTM super kit (mouse/rabbit) (Fuzhou Maixin Biotechnology Development Co., Ltd., China) was used to amplify the reaction, enhanced DAB chromogenic kit (Gene Technology Co., Ltd., China) was used for colour development and hematoxylin was used for re-staining. The antigen retrieval condition, dilution, incubation condition, catalog number, and lot number of these antibodies are shown in supplementary Table 1.

### Assessment of immunohistochemical staining

The results were determined by two senior pathologists using a double-blind method. The judging criteria were as follows: the sections were selected at random under low magnification of the microscope (100x) with 10 fields of view per section, and then scored under high magnification (400x) based on the degree of staining (0-3 points for negative staining, yellowish, light brown, dark brown, respectively), the range of positivity (1-4 points for 0-25%, 26-50%, 51-75%, and 76-100%), and the final scores can be multiplied together to a range of 0-12. The optimal threshold for each biomarker was calculated from the ROC curve and the Jorden index and is interpreted as positive or negative. The threshold values and subcellular location of these biomarkers are shown in supplementary Table 1.

### Statistical analysis

All the data of the enrolled cases were processed for statistical analysis using SPSS 25.0 software and Graphpad Prism 9 software. Because the total sample of 171 cases in this study was greater than 40, and all the theoretical frequencies were greater than 5, meeting the conditions of the Pearson’ s χ^2^ test. The Pearson’ s χ^2^ test was used to analyze the relationship between the expression of CAF, EMT, and CSC biomarkers and the clinicopathological factors of the cases. The study variables were all categorical and no covariance among them, therefore, Logistic regression was used to analyze the relationship between clinicopathological factors and biomarkers and pathological responses. Univariateand multivariate Cox proportional hazards model were used to examine the the relationship between all clinicopathological factors and the expression of CAF, EMT, and CSC markers and OS. Survival analysis was performed and plotted using Kaplan-Meier method, and differences in Kaplan-Meier curves were assessed using log-rank test. *P* < 0.05 means statistically significant, and the confidence interval (CI) was determined at the 95% level.

## Results

### The patient’s clinicopathological characteristics and pathological response assessment

#### Clinicopathological characteristics

Most patients were male (74.9%) and a greater proportion (78.9%) were younger than 65 years. Approximately half of the tumors were located in the lower third of the stomach (52.0%), while 24.0%, 18.1%, and 5.8% were localized in the middle third, upper third and gastroesophageal junction, and diffuse type respectively. The tumor diameter was greater than or equal to 5 cm in the majority of cases (62.0%). The predominant histological type was non-hypo adhesive adenocarcinoma (62.0%), with a slight predominance of intestinal type (52.0%) and only 19.9% of highly differentiated cases. 22.8% of cases showed lymphovascular invasion and 21.1% were associated with nerve invasion. The detailed clinicopathological information is shown in Table 1.

**Table 1:** Clinicopathological characteristics of the enrolled gastric cancer patients

Clinicopathological characteristic	N(%)
Gender	
Male	128 (74.9)
Female	43 (25.1)
Age (yr)	
<65	135 (78.9)
≥65	36 (21.1)
Tumor location	
Lower third	89 (52.0)
Middle third	41 (24.0)
UGEJ	31 (18.1)
Diffuse	10 (5.8)
Tumour size (cm)	
<5	65 (38.0)
≥5	106 (62.0)
ypT Stage	
0–2	39 (22.8)
3–4	132 (77.2)
ypN Stage	
0	63 (36.8)
1	31 (18.1)
2	29 (17.0)
3	48 (28.1)
ypTNM Stage	
I	32 (18.7)
II	43 (25.1)
III	96 (56.1)
Histological types	
Non-low-adherent adenocarcinoman	106 (62.0)
Low-adhesion adenocarcinoma	65 (38.0)
Lauren classification	
Intestinal	89 (52.0)
Diffuse or Mixed	82 (48.0)
Degree of differentiation	
Well	34 (19.9)
Moderate or Poor	137 (80.1)
Vascular or lymphatic invasion	
No	132 (77.2)
Yes	39 (22.8)
Nerve invasion	
No	135 (78.9)
Yes	36 (21.1)
Mandard TRG	
1–2	58 (33.9)
3–5	113 (66.1)

*UGEJ* Upper third and gastroesophageal junction

#### Pathological response assessment

**Fig. 1:**
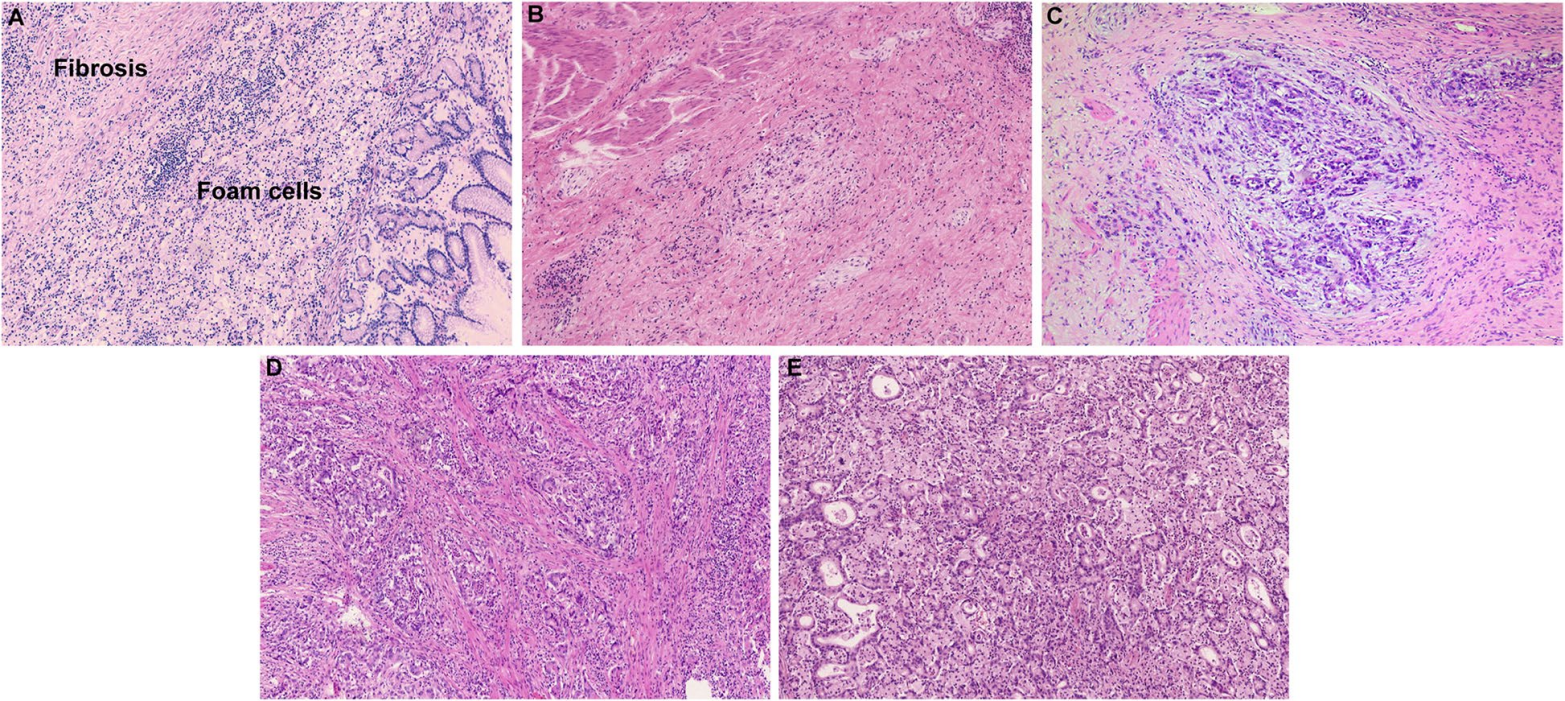
Examples of Mandard’s TRG (100x). **A** TRG 1 (complete regression, no evidence of residual tumor); **B** TRG 2 (only a few tumor cells remain, fibrosis is the main component); **C** TRG 3 (fibrosis and residual tumor, predominantly fibrosis); **D** TRG 4 (fibrosis and residual tumor, predominantly tumor); **E** TRG 5 (no evidence of regression, extensive residual tumor)

33.9% of patients were TRG 1-2. The OS between patients with TRG 1 and TRG 2 had no difference (*p* = 0.374), so they were classified into the NCT sensitive group (supplementary Fig. 1A); 66.1% of patients were TRG 3-5, the OS among patients with TRG 3, TRG 4, and TRG 5 had no difference (*p* = 0.560), so they were classified into the NCT resistant group (supplementary Fig. 1B). The OS between the sensitive group and the resistant group was significantly different (*p* < 0.001) (supplementary Fig. 1C). Typical pathological images of TRG grades 1-5 are shown in Fig. 1.

### Immunohistochemical staining results

**Fig. 2:**
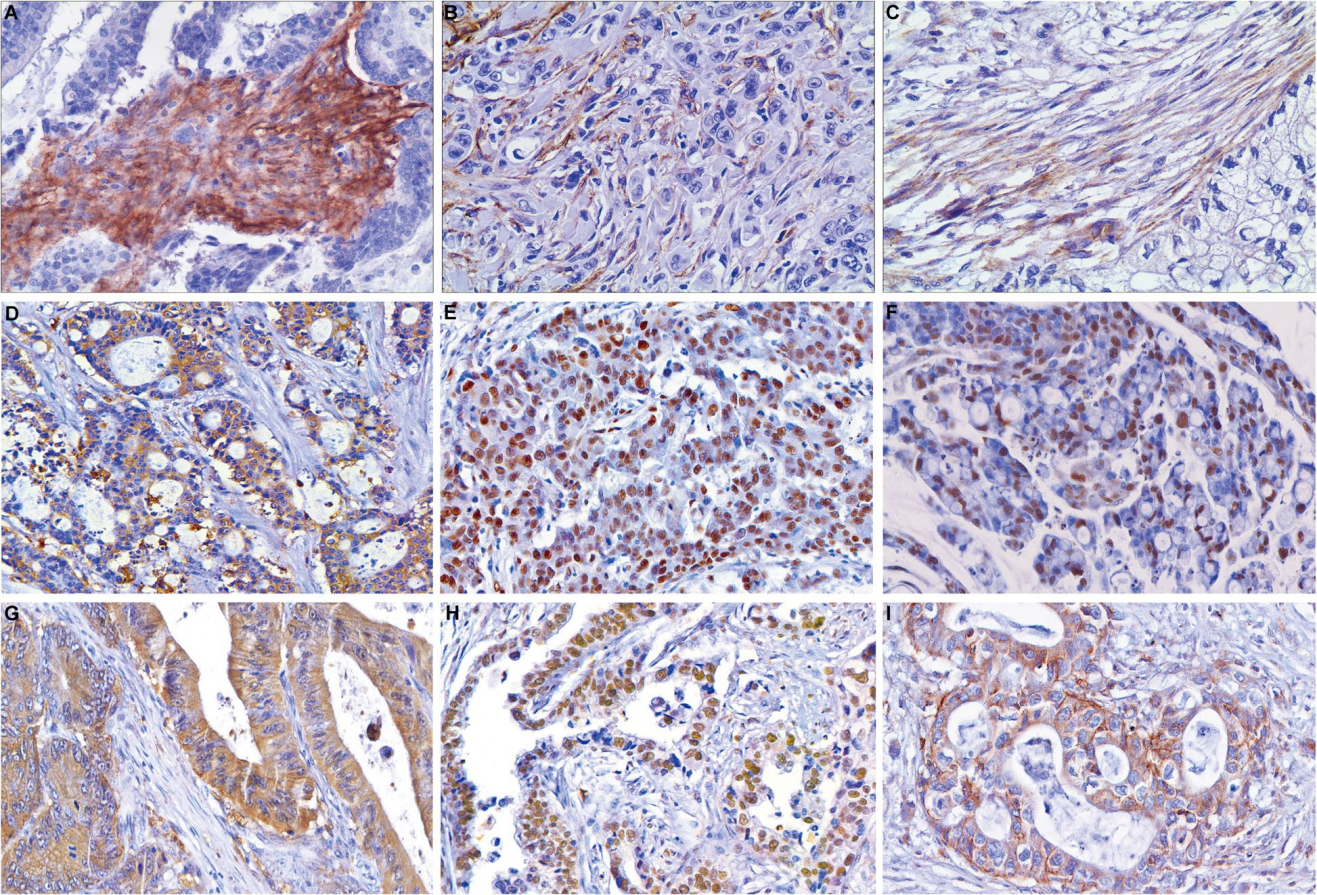
Representative examples of immunohistochemistry results (400x). **A** FAP expression in CAF cytoplasm; **B**. CD10 expression in CAF cell membranes and cytoplasm; **C** GPR77 expression in CAF cell membranes and cytoplasm; **D** N-cadherin expression in the cell membrane and cytoplasm of gastric cancer cells; **E** Snail1 expression in the nucleus of gastric cancer cells; **F** Twist1 expression in the nucleus of gastric cancer cells; **G** ALDH1 expression in the cell cytoplasm of gastric cancer cells; **H** LGR5 expression in the nucleus of gastric cancer cells; **I** CD44 expression in the cell membrane and cytoplasm of gastric cancer cells

Positive rates for CAF markers FAP, CD10, and GPR77 were 39.8%, 18.1%, and 12.3% respectively, and negative for fibroblasts in normal tissue. The EMT markers N-cadherin, Snail1, and Twist1 were positive in 37.4%, 28.7%, and 45.6% cases respectively, and negative in normal mucosal epithelial cells. Positive rates for the CSC markers ALDH1, CD44, and LGR5 were 64.3%, 46.2%, and 57.9% respectively, and negative in normal mucosal epithelial cells. The images of immunohistochemical staining are shown in Fig. 2.

### Relationship between CAF, EMT, and CSC biomarkers and clinicopathological characteristics

Using χ^2^ test we found that expression of markers of CAF, EMT, and CSC is associated with poorer clinicopathological factors. The relationships between the three CAF biomarkers, FAP, CD10, and GPR77, and the clinicopathological characteristics of the enrolled patients are shown in Table 2. The results showed that the higher expression of FAP, CD10, and GPR77 was significantly related to the higher grade of ypT, ypTNM, and Mandard TRG grade (*p* < 0.05). The higher FAP expression was correlated with larger tumor size (*p* = 0.002), higher stage of ypN (*p* = 0.034), poorer degree of differentiation (*p* = .031), and a greater tendency to lymphovascular invasion (*p* = 0.016), and nerve invasion (*p* = 0.029). Expression of CD10 was significantly associate with larger tumor size (*p* = 0.006), higher ypN stage (*p* = 0.034), and Lauren classification (diffuse or mixed type) (*p* = 0.006).

**Table 2:** The relationships between CAF, EMT, CSC biomarkers and clinicopathological characteristics

	**FAP**		***P***	**χ**^**2**^	**CD10**		***P***	**χ**^**2**^	**GPR77**		***P***	**χ**^**2**^	**N-cadherin**		***P***	**χ**^**2**^
	-	+			-	+			-	+			-	+		
Gender			0.140	2.180			0.716	0.132			0.221	1.500			0.260	1.270
Male	73	55			104	24			110	18			77	51		
Female	30	13			36	7			40	3			30	13		
Age (yr)			0.793	0.069			0.228	1.451			0.417	0.660			0.327	0.959
<65	82	53			113	22			117	18			87	48		
≥65	21	15			27	9			33	3			20	16		
Tumor location			0.725	1.319			0.339	3.363			0.255	4.057			0.930	0.449
Lower third	51	38			74	15			74	15			55	34		
Middle third	27	14			35	6			39	2			25	16		
UGEJ	18	13			22	9			28	3			21	10		
Diffuse	7	3			9	1			9	1			6	4		
Tumour size (cm)			**0.002**	10.049			**0.006**	7.695			0.152	2.049			0.159	1.985
<5	49	16			60	5			60	5			45	20		
≥ 5	54	52			80	26			90	16			62	44		
ypT Stage			**<0.001**	21.699			**0.016**	5.753			**0.035**	4.428			**0.004**	8.185
0–2	36	3			37	2			38	1			32	7		
3–4	67	65			103	29			112	20			75	57		
ypN Stage			**0.001**	16.972			**0.034**	8.689			0.118	5.872			**0.032**	8.834
0	49	14			57	6			60	3			46	17		
1	20	11			26	5			26	5			20	11		
2	14	15			19	10			23	6			19	10		
3	20	28			38	10			41	7			46	17		
ypTNM Stage			**<0.001**	24.251			**0.003**	11.832			**0.014**	8.543			**0.009**	9.376
I	30	2			30	2			31	1			26	6		
II	29	14			40	3			41	2			30	13		
III	44	52			70	26			78	18			51	45		
Histological types			0.961	0.002			0.255	1.296			0.333	0.938			0.666	0.187
Non-low-adhesion adenocarcinoman	64	42			84	22			95	11			65	41		
Low-adhesion adenocarcinoma	39	26			56	9			55	10			42	23		
Lauren classification			0.150	2.077			**0.006**	7.441			0.618	0.249			0.827	0.048
Intestinal	49	40			66	23			77	12			55	34		
Diffuse or Mixed	54	28			74	8			73	9			52	30		
Degree of differentiation			**0.031**	4.671			0.116	2.476			0.064	3.436			0.774	0.082
Well	26	8			31	3			33	1			22	12		
Moderate or Poor	77	60			109	28			117	20			85	52		
Vascular or lymphatic invasion			**0.016**	5.843			0.327	0.959			0.075	3.178			0.200	1.643
No	86	46			106	26			119	13			86	46		
Yes	17	22			34	5			31	8			21	18		
Nerve invasion			**0.029**	4.746			0.219	1.513			0.367	0.814			0.172	1.868
No	87	48			108	27			120	15			88	47		
Yes	16	20			32	4			30	6			19	17		
Mandard TRG			**<0.001**	43.852			**<0.001**	15.914			**0.003**	9.080			**0.004**	8.447
1–2	55	3			57	1			57	1			45	13		
3–5	48	65			83	30			93	20			62	51		

	**Snail1**		***P***	**χ**^**2**^	**Twist1**		***P***	**χ**^**2**^	**ALDH1**		***P***	**χ**^**2**^	**LGR5**		***P***	**χ**^**2**^	**CD44**		***P***	**χ**^**2**^
	-	+			-	+			-	+			-	+			-	+		
Gender			0.092	2.838			0.624	0.241			0.086	2.941			0.301	1.068			0.760	0.094
Male	87	41			71	57			41	87			51	77			68	60		
Female	35	8			22	21			20	23			21	22			24	19		
Age (yr)			0.337	0.923			0.874	0.025			0.951	0.004			0.484	0.490			0.539	0.377
<65	94	41			73	62			48	87			55	80			71	64		
≥65	28	8			20	16			13	23			17	19			21	15		
Tumor location			0.906	0.559			0.657	1.612			0.258	4.028			0.226	4.349			0.876	0.690
Lower third	62	27			49	40			37	52			43	46			50	39		
Middle third	30	11			21	20			13	28			15	26			20	21		
UGEJ	22	9			19	12			7	24			9	22			17	14		
Diffuse	8	2			4	6			4	6			5	5			5	5		
Tumour size (cm)			0.360	0.837			**0.002**	9.315			0.789	0.071			0.840	0.041			0.112	2.526
<5	49	16			45	20			24	41			28	37			40	25		
≥ 5	73	33			48	58			37	69			44	62			52	54		
ypT Stage			**0.037**	4.352			**<0.001**	25.461			0.240	1.380			**0.039**	4.241			0.067	3.364
0–2	33	6			35	4			17	22			22	17			26	13		
3–4	89	43			58	74			44	88			50	82			66	66		
ypN Stage			0.606	1.842			0.060	7.399			0.203	4.601			0.986	0.147			0.382	3.066
0	48	15			42	21			27	36			26	37			32	31		
1	23	8			17	14			13	18			14	17			21	10		
2	19	10			14	15			9	20			12	17			14	15		
3	32	16			20	28			12	36			20	28			25	23		
ypTNM Stage			0.193	3.294			**<0.001**	23.236			0.194	3.279			0.198	3.243			0.307	2.360
I	27	5			29	3			15	17			18	14			21	11		
II	29	14			24	19			17	26			17	26			23	20		
III	66	30			40	56			29	67			37	59			48	48		
Histological types			0.107	2.598			0.669	0.183			0.551	0.355			0.906	0.014			0.745	0.106
Non-low-adhesion adenocarcinoman	71	35			59	47			36	70			45	61			56	50		
Low-adhesion adenocarcinoma	51	14			34	31			25	40			27	38			36	29		
Lauren classification			**0.011**	6.442			0.460	0.546			0.576	0.312			0.443	0.588			**0.035**	4.466
Intestinal	56	33			46	43			30	59			35	54			41	48		
Diffuse or Mixed	66	16			47	35			31	51			37	45			51	31		
Degree of differentiation			0.753	0.099			**0.034**	4.491			0.251	1.319			**0.009**	6.729			0.512	0.431
Well	25	9			24	10			15	19			21	13			20	14		
Moderate or Poor	97	40			69	68			46	91			51	86			72	65		
Vascular or lymphatic invasion			0.462	0.541			0.419	0.654			0.137	2.216			0.103	2.663			0.276	1.189
No	96	36			74	58			51	81			60	72			74	58		
Yes	26	13			19	20			10	29			12	27			18	21		
Nerve invasion			0.777	0.081			0.827	0.048			0.650	0.206			0.660	0.194			0.539	0.377
No	97	38			74	61			47	88			58	77			71	64		
Yes	25	11			19	17			14	22			14	22			21	15		
Mandard TRG			**0.006**	7.410			**<0.001**	35.825			**0.033**	4.527			**0.002**	9.821			**0.012**	6.379
1–2	49	9			50	8			27	31			34	24			39	19		
3–5	73	40			43	70			34	79			38	75			53	60		

*UGEJ* Upper third and gastroesophageal junction

The relationships between the three EMT biomarkers N-cadherin, Snail1, and Twist1, and the clinicopathological characteristics are shown in Table 2. The results showed that higher expression of N-cadherin, Snail1, and Twist1 all correlated significantly with higher TRG grade (*p* = 0.004, 0.006, < 0.001). Higher expression of both N-cadherin and Twist1 correlated significantly with higher stage of ypT (*p* = 0.004, < 0.001) and ypTNM (*p* = 0.009, < 0.001). In addition, higher expression of N-cadherin was also significantly associated with higher ypN (*p* = 0.032). Higher expression of Twist1 was strongly related to larger tumor size (*p* = 0.002) and poorer degree of differentiation (*p* = 0.034). Higher expression of Snail1 was significantly related to higher ypT (*p* = 0.037) and Lauren classification (diffuse or mixed type) (*p* = 0.011).

The relationships between the three CSC biomarkers, ALDH1, CD44, and LGR5, and the clinicopathological characteristics are shown in Table 2. The analysis results suggested that higher expression of ALDH1, CD44, and LGR5 was significantly correlated with higher the grade of TRG grading (*p* = 0.033, 0.002, and 0.012). In addition, higher expression of CD44 was significantly related to Lauren classification (diffuse or mixed type) (*p* = 0.035). Higher expression of LGR5 was significantly associated with higher ypT stage (*p* = 0.039) and poorer degree of differentiation (*p* = 0.009).

### Relationship between CAF markers and EMT, CSC markers

**Fig. 3:**
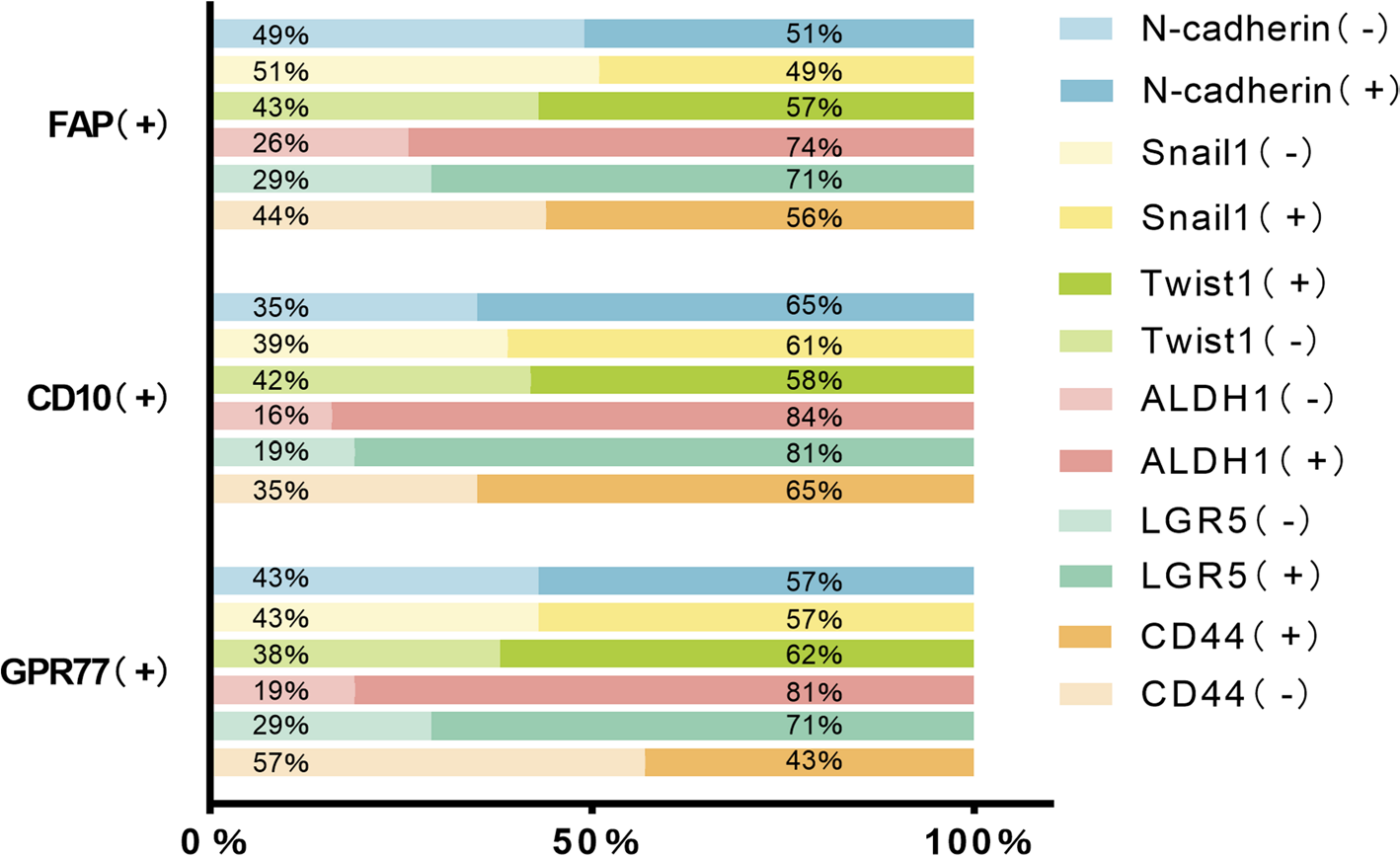
Association of CAF markers with EMT and CSC marker expression. Expression of EMT and CSC markers in FAP, CD10, and GPR77-positive cases

The three markers of CAF were specifically correlated with EMT and CSC markers as shown in Table 3. Figure 3 shows the proportion of cases with positive expression of EMT and CSC markers among those with positive expression of FAP, CD10, and GPR77 in CAFs. Expression of FAP, CD10, and GPR77 were closely correlated with EMT markers. FAP expression in CAFs was positively related to N-cadherin (*p* = 0.002), Snail1 (*p* < 0.001), and Twist1 (*p* = 0.012); CD10 expression was positively associated with N-cadherin (*p* = 0.001) and Snail1 (*p* < 0.001); GPR77 expression was positively correlated with N-cadherin (*p* = 0.046) and Snail1 (*p* = 0.002).

**Table 3:** Relationship between CAF markers and EMT, CSC markers

	**FAP**		***P***	**χ**^**2**^	**CD10**		***P***	**χ**^**2**^	**GPR77**		***P***	**χ**^**2**^
	-	+			-	+			-	+		
N-cadherin			**0.002**	9.507			**0.001**	11.865			**0.046**	3.974
-	74	33			96	11			98	9		
+	29	35			44	20			52	12		
Snail1			**<0.001**	21.812			**<0.001**	19.726			**0.002**	9.503
-	87	35			110	12			113	9		
+	16	33			30	19			37	12		
Twist1			**0.012**	6.271			0.124	2.366			0.110	2.561
-	64	29			80	13			85	8		
+	39	39			60	18			65	13		
ALDH1			**0.041**	4.166			**0.012**	6.302			0.089	2.883
-	43	18			56	5			57	4		
+	60	50			84	26			93	17		
LGR5			**0.006**	7.462			**0.005**	8.040			0.180	1.799
-	52	20			66	6			66	6		
+	51	48			74	25			84	15		
CD44			**0.039**	4.259			**0.024**	5.111			0.743	0.108
-	62	30			81	11			80	12		
+	41	38			59	20			70	9

The expression of FAP and CD10 were closely correlated with CSC markers. The expression of FAP was positively related to ALDH1 (*p* = 0.041), LGR5 (*p* = 0.006), and CD44 (*p* = 0.039); the expression of CD10 was positively correlated with ALDH1 (*p* = 0.012), LGR5 (*p* = 0.005), and CD44 (*p* = 0.024); while no significant correlation was found between the expression of GPR77 and CSC markers.

### Predictive value of biomarkers for pathological response

CAF biomarkers FAP (OR=24.826, *p* < 0.001), CD10 (OR=20.602, *p* = 0.003), GPR77 (OR=12.258, *p* = 0.016), EMT marker N-cadherin (OR=2.847, *p* = 0.004), Snail1 (OR=2.983, *p* = 0.008), Twist1 (OR=10.174, *p* < 0.001), and CSC markers ALDH1 (OR=2.024, *p* = 0.035), LGR5 (OR=2.796, *p* = 0.002), and CD44 (OR=2.324, *p* = 0.012) were strongly related to poorer pathological response. For clinicopathological characteristics, the larger the tumor, the higher the ypT, ypN, and ypTNM stages, the worse the pathological response (all *p* < 0.001). In addition, histological type (low-adhesion adenocarcinoma) (OR=2.275, *p* = 0.020), poorer degree of differentiation (OR=4.336, *p* < 0.001), vascular invasion (OR=3.575, *p* = 0.008) and neural invasion (OR=4.007, *p* = 0.007) were also important factors influencing the pathological response (Table 4).

**Table 4:** Univariable and multivariable analysis of the pathological response

	**Univariable analysis**		**Multivariable analysis**	
	**OR (95%CI)**	***P***	**OR (95%CI)**	***P***
Gender (Female)	0.721 (0.353, 1.474)	0.370		
Age(≥65yr)	0.760 (0.355, 1.626)	0.479		
Tumor location		0.444		
Lower third	1			
Middle third	0.892 (0.344, 2.313)	0.814		
UGEJ	1.450 (0.618, 3.401)	0.393		
Diffuse	2.526 (0.457, 13.964)	0.288		
Tumour size (≥5cm)	6.052 (3.052, 12.101)	<0.001	4.397 (1.190, 16.249)	**0.026**
ypT(3-4)	30.600 (10.840, 86.379)	**<0.001**	6.260 (1.124, 34.867)	**0.036**
ypN		**<0.001**		0.320
0	1		1	
1	1.429 (0.600, 3.404)	0.420	0.454 (0.044, 4.663)	0.506
2	6.452 (2.012, 20.690)	**0.002**	1.428 (0.065, 31.308)	0.821
3	4.473 (1.861, 10.753)	**<0.001**	0.228 (0.012, 4.397)	0.327
ypTNM (III)	6.364 (3.147, 12.869)	**<0.001**	1.891 (0.139, 25.676)	0.632
Histological types	2.275 (1.135, 4.559)	**0.020**	2.231 (0.598, 8.317)	0.232
Lauren classification	1.663 (0.874, 3.162)	0.121		
Degree of differentiation	4.336 (1.986, 9.599)	**<0.001**	1.718 (0.399, 7.398)	0.468
Vascular or lymphatic invasion	3.575 (1.400, 9.127)	**0.008**	3.104 (0.518, 18.588)	0.215
Nerve invasion	4.007 (1.466, 10.956)	**0.007**	1.838 (0.351, 9.622)	0.471
CAF markers				
FAP (+)	24.826 (7.326, 84.128)	**<0.001**	16.905 (2.468,115.810)	**0.004**
CD10 (+)	20.602 (2.731, 155.415)	**0.003**	19.022 (0.506,715.246)	0.111
GPR77 (+)	12.258 (1.601, 93.825)	**0.016**	3.365 (0.131, 86.710)	0.464
EMT markers				
N-cadherin (+)	2.847 (1.386, 5.849)	**0.004**	0.512 (0.125, 2.093)	0.351
Snail1 (+)	2.983 (1.329, 6.697)	**0.008**	0.521 (0.108, 2.510)	0.416
Twist1 (+)	10.174 (4.404, 23.506)	**<0.001**	14.238 (3.020, 67.130)	**0.001**
CSC markers				
ALDH1 (+)	2.024 (1.052,3.892)	**0.035**	0.844 (0.237, 3.005)	0.794
LGR5 (+)	2.796 (1.456,5.368)	**0.002**	1.861 (0.404, 8.583)	0.426
CD44 (+)	2.324 (1.200, 4.501)	**0.012**	0.827 (0.205, 3.333)	0.790

*UGEJ* Upper third and gastroesophageal junction

Factors of significance in the univariate analysis, as well as CAF, EMT, and CSC biomarkers, were included in the multivariable analysis for the pathological response. The results suggested that largerer tumor size (OR=4.397, *p* = 0.026), higher ypT stage (OR=6.260, *p* = 0.036), higher expression of FAP in CAF (OR=16.905, *p* = 0.004), and higher expression of Twist1 (OR=14.238, *p* = 0.001) were independent predictors for the pathological response, respectively (Table 4).

### Prognostic value of biomarkers

**Fig. 4:**
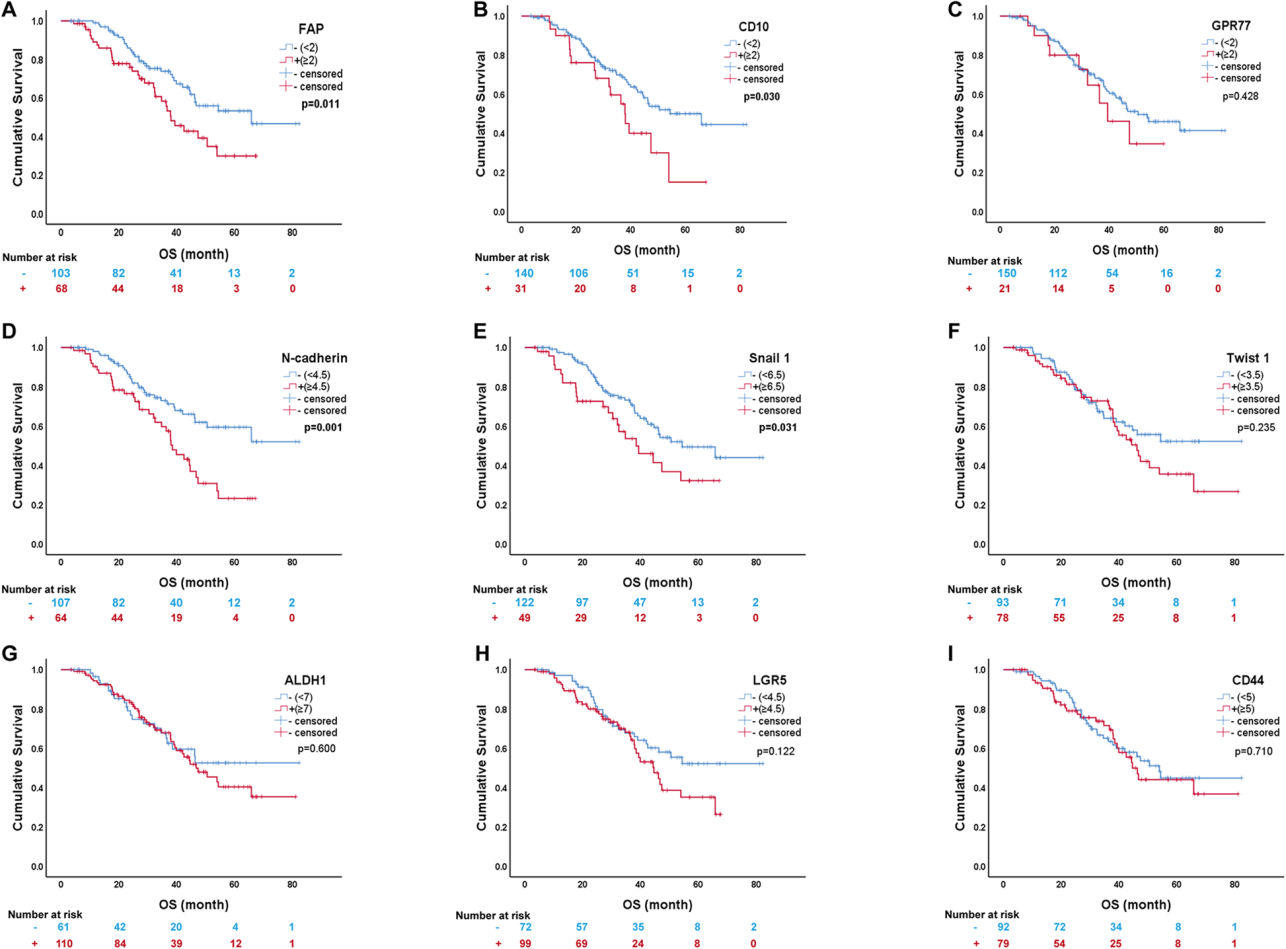
Survival analysis of expression of 9 biomarkers in 171 cases by Kaplan-Meier method. **A** FAP; **B** CD10; **C** GPR77; **D** N-cadherin; **E** Snail1; **F** Twist1; **G** ALDH1; **H** LGR5; **I** CD44(*p* = 0.71). FAP (*p* = 0.011), CD10 (*p* = 0.030), N-cadherin (*p* = 0.001), and Snail1 (*p* = 0.031) were significantly related to OS

The survival curves of CAF, EMT, and CSC biomarkers are shown in Fig. 4. In the univariable analysis for OS, the expression of FAP (OR=1.843, *p* = 0.013) and CD10 (OR=1.832, *p* = 0.032) in CAF, as well as the expression of the EMT markers N-cadherin (OR=2.158, *p* = 0.002) and Snail1 (OR=1.735, *p* = 0.033), were important factors influencing patient prognosis. Higher expression of these biomarkers was associated with poorer prognosis. For clinicopathological factors, tumor site (OR=4.539, *p* = 003), tumor size (OR=3.092, *p* < 0.001), ypT (OR=7.331, *p* < 0.001), ypN (OR=3.256, *p* < 0.001), ypTNM staging (OR=4.442, *p* < 0.001), and Lauren type (OR=1.925, *p* < 0.009), degree of differentiation (OR=2.733, *p* = 0.008), vascular invasion (OR=1.901, *p* = 0.016), and Mandard TRG grade (OR=2.861, *p* = 0.001) were correlated with the prognosis (Table 5).

**Table 5:** Univariable and multivariable analysis for overall survival

	**Univariable analysis**		**Multivariable analysis**	
	**HR (95%CI)**	***P***	**HR (95%CI)**	***P***
Gender (Female)	1.474 (0.852, 2.550)	0.165		
Age(≥65yr)	1.402 (0.816, 2.406)	0.221		
Tumor location		**0.008**		**0.032**
Lower third	1		1	
Middle third	1.586 (0.898, 2.800)	0.112	1.756 (0.915, 3.369)	0.091
UGEJ	0.712 (0.314, 1.617)	0.417	0.437 (0.159, 1.198)	0.108
Diffuse	3.232 (1.480, 7.055)	**0.003**	2.220 (0.881, 5.593)	0.091
Tumour size (≥5cm)	3.092 (1.684, 5.680)	**<0.001**	1.808 (0.812, 4.024)	0.147
ypT (3-4)	7.331 (2.659, 20.215)	**<0.001**	3.671 (0.995, 13.564)	0.051
ypN (2-3)	3.256 (1.969, 5.385)	**<0.001**	1.930 (0.709, 5.257)	0.198
ypTNM (III)	4.442 (2.456, 8.036)	**<0.001**	1.816 (0.854, 3.903)	0.126
Histological types	1.167 (0.716, 1.904)	0.535		
Lauren classification	1.925 (1.178, 3.144)	**0.009**	1.576 (0.814, 3.054)	0.177
Degree of differentiation	2.733 (1.303, 5.731)	**0.008**	1.679 (0.682, 4.130)	0.259
Vascular or lymphatic invasion	1.901 (1.126, 3.210)	**0.016**	1.141 (0.571, 2.280)	0.708
Nerve invasion	1.256 (0.716, 2.206)	0.427		
Mandard TRG (3-5)	2.861 (1.557, 5.260)	**0.001**	0.669 (0.297, 1.506)	0.332
CAF markers				
FAP (+)	1.843 (1.139, 2.983)	**0.013**	1.465 (0.652, 3.292)	0.356
CD10 (+)	1.832 (1.053, 3.189)	**0.032**	0.920 (0.436, 1.944)	0.827
GPR77 (+)	1.329 (0.657, 2.690)	0.429	0.697 (0.291, 1.669)	0.418
EMT markers				
N-cadherin (+)	2.158 (1.334, 3.490)	**0.002**	1.908 (1.057, 3.443)	**0.032**
Snail1 (+)	1.735 (1.047, 2.876)	**0.033**	2.081 (1.082, 4.004)	**0.028**
Twist1 (+)	1.335 (0.827, 2.157)	0.237	0.634 (0.357, 1.128)	0.121
CSC markers				
ALDH1 (+)	1.148 (0.685, 1.925)	0.601	0.681 (0.367, 1.265)	0.224
LGR5 (+)	1.473 (0.899, 2.413)	0.124	1.685 (0.916, 3.100)	0.093
CD44 (+)	1.095 (677, 1.772)	0.710	0.627 (0.334, 1.179)	0.147

*UGEJ* Upper third and gastroesophageal junction

In multivariable analysis, CAF, EMT, CSC biomarkers, and other statistically significant factors were included for prognosis. Tumor site (*p* = 0.032), expression of the EMT marker N-cadherin (OR=1.908, *p* = 0.032) and Snail1 (OR=2.081, *p* = 0.028) were independent prognostic factors for OS (Table 5).

## Discussion

With the current development of treatment technology, the combination of NCT and surgery has propelled gastric cancer treatment to a new level. However, for patients who have undergone NCT, there are still some patients whose pathological regression is not satisfactory. CAFs in the tumor microenvironment have been reported to secrete chemokines, which confer EMT and CSC traits to tumor cells, ultimately leading to chemoresistance [37–39]. This provides us with new research ideas to overcome NCT resistance in gastric cancer.

In this study, we analyzed the relationship between the expression CAF, EMT, CSC biomarkers, and clinicopathological characteristics in paraffin-embedded specimens from patients undergoing surgical resection after NCT; and analyzed the correlation between CAF markers and EMT, CSC markers, and the predictive value of these biomarkers for pathological response and OS. Our results revealed that the more CAFs expressing FAP, CD10, and GPR77, the worse the clinicopathological factors of gastric cancer, including higher ypT stage, ypTNM stage, ypN stage, poorer tumor differentiation, and more likely to develop lymphatic vascular invasion and nerve invasion. These results all suggest that CAF subpopulations expressing FAP, CD10, and GPR77 can promote tumor growth, invasion, and metastasis, suggesting they are cancer-promoting CAF markers. Our findings are consistent with those of Gong et al, who found that FAP^+^CAFs promote metastasis of lobulated breast tumors [40]. In addition, FAP^+^CAFs are associated with the invasion of high-grade invasive uroepithelial carcinoma of the bladder [22], invasion and proliferation of prostate cancer [18], and infiltration depth of esophageal cancer [19]. In renal clear cell carcinoma, FAP^+^CAFs are strongly associated with larger tumor diameter (>7 cm), higher grade (G3/4), higher T-stage (pT3/4), tumor necrosis, sarcomatoid transformation, and early lymph node metastasis [20]. In breast cancer, CD10^+^CAFs are associated with ER-negative invasive breast cancer, while CD10^-^CAFs are correlated with luminal-type invasive breast cancer [23]. In uroepithelial carcinoma, CD10 expression in CAF is significantly related to poorer clinicopathological factors, such as squamous differentiation of tumor cells, lymph node metastasis and necrosis [25]. These findings support that FAP, CD10, and GPR77-labeled CAF has cancer-promoting properties.

TRG is a system for assessing the amount of residual tumor, that can be used to evaluate therapeutic efficacy and predict prognosis [41, 42]. The Mandard TRG scoring system is routinely used clinically to evaluate the efficacy of chemotherapy. Our study found that the CAF markers FAP, CD10, and GPR77 were closely associated with the Mandard TRG; the more FAP, CD10, and GPR77 were expressed in CAF, the higher the Mandard TRG score, suggesting that all three labeled CAFs were associated with NCT resistance in gastric cancer. EMT and CSC are important mechanisms of chemotherapy resistance. Our study found that the expression of all three CAF markers was positively correlated with that of EMT and CSC markers, that was closely related to TRG score. These results suggest that CAF expressing FAP, CD10, GPR77 may lead to drug resistance by inducing tumors to develop EMT or CSC in gastric cancer. It has been found that FAP^+^CAFs promote EMT of gastric cancer cells via the Wnt/β-catenin signaling pathway [16]. In colorectal cancer, FAP^+^CAF secretes TGFβ, which activates the classical TGFβ signaling pathway and induces transcriptional regulation of Snail1 and Twist1 target genes, leading to EMT in cancer cells [43]. The same findings are also seen in bladder cancer [44] and lung cancer [45]. Furthermore, a subpopulation of CAF with high CD10 and GPR77 expression is associated with chemoresistance in patients with breast cancer and lung cancer. CD10^+^GPR77^+^ CAFs provide constant paracrine IL-6 and IL-8 through sustained nuclear factor kappa-B (NF-$\mathrm\kappa$B) signaling maintained by p65 phosphorylation and acetylation, forming ecological niche that protects CSC from chemotherapy-induced cell death [26]. All these findings support our conclusion that CAF expressing FAP, CD10, and GPR77 may lead to drug resistance through the induction of EMT or CSC in gastric cancer cells.

In addition, the relationship between CAF, EMT, CSC biomarkers, clinicopathological factors, and prognosis was analyzed in this study. In univariate analysis, TRG grading, CAF markers (FAP, CD10), and EMT markers (Snail1, N- cadherin) were related to poorer prognosis in patients with gastric cancer. This may be caused by the malignant biological behavior due to CAF-induced EMT pathway. In colorectal cancer [20, 46], ovarian cancer [21], non-small cell lung cancer [17], high-grade invasive uroepithelial carcinoma of the bladder [22], pancreatic cancer [47], and melanoma [48], FAP^+^CAFs also predict a poorer prognosis for patients. Applying multiplex immunofluorescence, Sun et al. found that low FAP protein expression in CAFs was associatied with a significantly better OS and DFS than high FAP protein expression in patients with GC [49]. High CD10-expressing CAF subpopulations are related to poor survival in patients with breast and lung cancer [26]. In multifactorial analysis, only tumor site, Snail1, and N-cadherin were independent prognostic prognostic factors, while TRG grading, CAF marker FAP and CD10 were not. It may be due to the fact that CAF itself does not directly affect prognosis, but indirectly affects prognosis by inducing EMT in tumor cells; and it may also be caused by the co-linearity between Snail1, N-cadherin and TRG grading and CAF markers.

This was a retrospective study from a single institution, which may result in bias. Secondly, this study was restricted to the protein level in vivo and did not perform in-depth exploration of molecular mechanisms in vitro. However, we focused on a unique group of patients with gastric cancer to validate the value of CAF biomarkers for clinical application and to explore the relationship of these biomarkers with chemo-resistance and prognosis; we also provided a preliminary investigation of its mechanisms. These results may contribute to the mechanisms of resistance to NCT in gastric cancer. Future research should be done to improve the understanding of the chemo-resistant mechanisms.

In conclusion, FAP, CD10, and GPR77 labeled CAF subgroup may promote gastric cancer progression, and lead to NCT resistance and poor prognosis by inducing EMT and CSC of gastric cancer cells in locally advanced gastric cancer patients.

## Supplementary Information

**Additional file 1: Supplementary Table 1.** Information about biomarkers.

**Additional file 2: Supplementary Figure1.** Kaplan-Meier curves for OS of patients with different TRG. A. The OS between patients with TRG 1 and TRG 2 had no difference (*p* = 0.374); B. the OS among patients with TRG 3, TRG 4, and TRG 5 had no difference (*p* = 0.560); C. The OS between patients with TRG 1-2 and TRG 3-5 was significantly different (*p* < 0.001).

## Data Availability

All data included in this study are available upon request by contact with the corresponding author.
