# Supplementary material for: FAP, CD10, and GPR77-labeled CAFs cause neoadjuvant chemotherapy resistance by inducing EMT and CSC in gastric cancer
Source: BMC Cancer. 2023 Jun 5;23:507. doi: 10.1186/s12885-023-11011-0 (PMC10240717; doi:10.1186/s12885-023-11011-0)
Supplement: Supplementary file 1 — Additional file 1: Supplementary Table 1. Information about biomarkers. [file 12885_2023_11011_MOESM1_ESM.docx]

| **Supplementary Table 1** Information about biomarkers | | | | | | | | |
| --- | --- | --- | --- | --- | --- | --- | --- | --- |
| **Antibodies** | **Manufacturers** | **catalog number** | **lot number** | **Antigen retrieval condition** | **Dilution** | **Incubation condition** | **Cut-off value** | **Subcellular Location** |
| FAP | Boster | [BM5121](https://www.boster.com.cn/home/product/anti-fap-antibody_bm5121.html) | BST17535121 | pressure cooker；citrate buffer pH6.0 | 1：400 | overnight at 4°C | 2 | Cytoplasm |
| CD10 | Maixin | MAB-0668-6 | 2008260668d | pressure cooker；EDTA buffer pH9.0 | Ready-to-Use Antibodies | overnight at 4°C | 2 | Cell membrane and cytoplasm |
| GPR77 | Abcam | ab116709 | GR196032-15 | pressure cooker；citrate buffer pH6.0 | 1：400 | overnight at 4°C | 2 | Cell membrane and cytoplasm |
| N-cadherin | Affinity | AF4039 | 40x8956 | pressure cooker；EDTA buffer pH9.0 | 1：400 | overnight at 4°C | 4.5 | Cell membrane and cytoplasm |
| Snail 1 | Affinity | AF6032 | 78m6660 | pressure cooker；citrate buffer pH6.0 | 1：400 | overnight at 4°C | 6.5 | Nucleus |
| Twist 1 | Affinity | AF4009 | 53t0783 | pressure cooker；citrate buffer pH6.0 | 1：400 | overnight at 4°C | 3.5 | Nucleus |
| ALDH1 | Boster | M01392-2 | 16T15B4C3 | pressure cooker；EDTA buffer pH9.0 | 1：800 | overnight at 4°C | 7 | Cytoplasm |
| LGR5 | Affinity | DF2816 | 21e8518 | pressure cooker；EDTA buffer pH9.0 | 1：200 | overnight at 4°C | 4.5 | Nucleus |
| CD44 | Boster | PB9333 | 2P771BP71① | pressure cooker；EDTA buffer pH9.0 | 1：800 | overnight at 4°C | 5 | Cell membrane and cytoplasm |
