# Supplementary figures and images for: FAP, CD10, and GPR77-labeled CAFs cause neoadjuvant chemotherapy resistance by inducing EMT and CSC in gastric cancer
Source: BMC Cancer. 2023 Jun 5;23:507. doi: 10.1186/s12885-023-11011-0 (PMC10240717; doi:10.1186/s12885-023-11011-0)

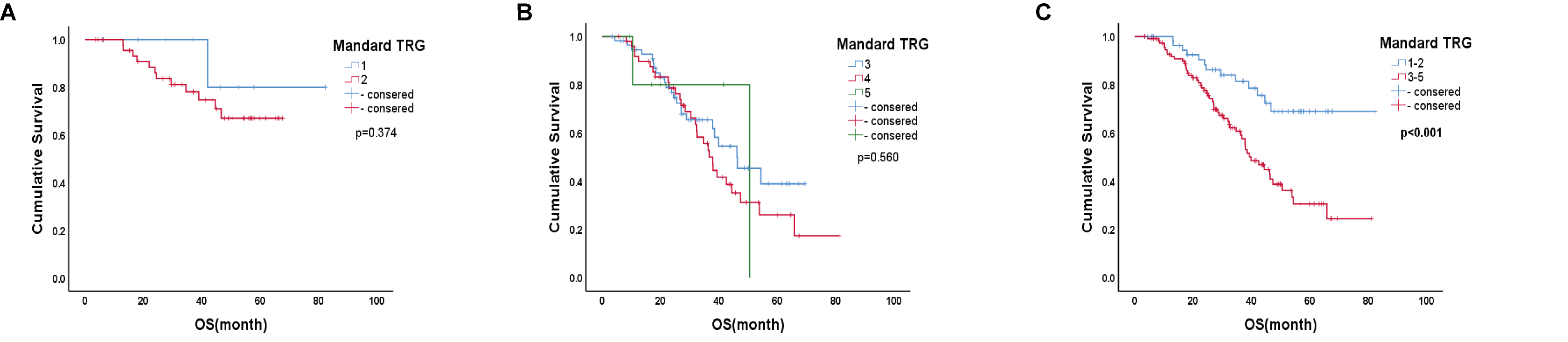

Supplement: Supplementary file 2 — Additional file 2: Supplementary Figure1. Kaplan-Meier curves for OS of patients with different TRG. A. The OS between patients with TRG 1 and TRG 2 had no difference (p = 0.374); B. the OS among patients with TRG 3, TRG 4, and TRG 5 had no difference (p = 0.560); C. The OS between patients with TRG 1-2 and TRG 3-5 was significantly different (p < 0.001). [file 12885_2023_11011_MOESM2_ESM.tif]
